# Inequities in child survival in Nigerian communities during the Sustainable Development Goal era: insights from analysis of 2016/2017 Multiple Indicator Cluster Survey

**DOI:** 10.1186/s12889-020-09672-8

**Published:** 2020-10-27

**Authors:** Daniel Adedayo Adeyinka, Nazeem Muhajarine, Pammla Petrucka, Elon Warnow Isaac

**Affiliations:** 1 Department of Community Health and Epidemiology, College of Medicine, University of Saskatchewan, Saskatoon, SK S7N 5E5 Canada; 2 Department of Public Health, Federal Ministry of Health, Abuja, Nigeria; 3 Saskatchewan Population Health and Evaluation Research Unit, Saskatoon, Saskatchewan Canada; 4 College of Nursing, University of Saskatchewan, Saskatoon, Canada; 5 Department of Paediatrics, College of Medical Sciences, Gombe State University, Gombe, Nigeria

**Keywords:** Social determinants of health, Sustainable development goals, Neonatal mortality, Post-neonatal mortality, Child mortality, Child survival, Multilevel analysis, Multinomial regression, Nigeria

## Abstract

**Background:**

Child survival is a major concern in Nigeria, as it contributes 13% of the global under-five mortalities. Although studies have examined the determinants of under-five mortality in Nigeria, the comparative roles of social determinants of health at the different stages of early childhood development have not been concurrently investigated. This study, therefore, aimed to identify the social determinants of age-specific childhood (0–59 months) mortalities, which are disaggregated into neonatal mortality (0–27 days), post-neonatal mortality (1–11 months) and child mortality (12–59 months), and estimate the within-and between-community variations of mortality among under-five children in Nigeria. This study provides evidence to guide stakeholders in planning for effective child survival strategies in the Nigerian communities during the Sustainable Development Goals era.

**Methods:**

Using the 2016/2017 Nigeria Multiple Indicator Cluster Survey, we performed multilevel multinomial logistic regression analysis on data of a nationally representative sample of 29,786 (weighted = 30,960) live births delivered 5 years before the survey to 18,497 women aged 15–49 years and nested within 16,151 households and 2227 communities.

**Results:**

Determinants of under-five mortality differ across the neonatal, post-neonatal and toddler/pre-school stages in Nigeria. Unexpectedly, attendance of skilled health providers during delivery was associated with an increased neonatal mortality risk, although its effect disappeared during post-neonatal and toddler/pre-school stages. Also, our study found maternal-level factors such as maternal education, contraceptive use, maternal wealth index, parity, death of previous children, and quality of perinatal care accounted for high variation (39%) in childhood mortalities across the communities. The inclusion of other compositional and contextual factors had no significant additional effect on childhood mortality risks across the communities.

**Conclusion:**

This study reinforces the importance of maternal-level factors in reducing childhood mortality, independent of the child, household, and community-level characteristics in the Nigerian communities. To tackle childhood mortalities in the communities, government-led strategies should prioritize implementation of community-based and community-specific interventions aimed at improving socioeconomic conditions of women. Training and continuous mentoring with adequate supervision of skilled health workers must be ensured to improve the quality of perinatal care in Nigeria.

**Supplementary information:**

**Supplementary information** accompanies this paper at 10.1186/s12889-020-09672-8.

## Background

As a critical element of socioeconomic and health indicators of a country, child survival has consistently been prominent in the United Nations suite of indicator frameworks [1]. The Sustainable Development Goal 3 (SDG-3) aims to reduce under-five mortality rates (U5MR) to 25 deaths per 1000 live births and neonatal mortality rates (NMR) to 12 deaths per 1000 live births by 2030 [2, 3]. Despite concerted global efforts in curtailing the preventable childhood deaths, progress in sub-Saharan Africa is lagging behind that of developed countries [1]. Specifically, childhood mortality remains a major social and public health problem in Nigeria, making the country the second largest contributor to under-five deaths globally [1]. Like many countries in the region, Nigeria could not achieve the childhood mortality targets of Millennium Development Goals (MDG)—aimed at reducing U5MR by two-thirds from 1990 to 2015 [1]. NMR and U5MR in Nigeria are unacceptably high—32.9 deaths per 1000 live births and 100.2 deaths per 1000 live births in 2017, respectively [1]. These are 1.8 and 2.6 times higher than the global NMR and U5MR, respectively [1].

For decades, some child survival initiatives for reducing under-five deaths have been non-specific, ineffectively addressing the varying roles of social determinants of health on mortality risks across the different stages of a child’s early development i.e., neonatal (0–27 days), post-neonatal (1–11 months) and toddler/pre-school (12–59 months) [4, 5]. Previous studies conducted in Nigeria have identified the micro-level/compositional (i.e., child and maternal and household-level factors) associated with neonatal, infant, and under-five mortality. Generally, male child, low level of parental education, poverty, previous short birth intervals (< 2 years), teenage motherhood, inadequate access to maternal healthcare services, and poor sanitation are associated with under-five mortality risk [6–19]. Despite these findings, literature remains limited regarding the effects of some individual compositional, and community contextual factors. Some of these available studies in Nigeria have provided contradictory results. For example, Ezeh et al. [17] and Morakinyo et al. [13] have linked maternal education to lower mortality risk among under-five children. Counterintuitively, Kayode et al. [20], Yaya et al. [21], and Akinyemi et al. [22] reported that maternal education had no significant effect on child survival. While studies [13, 17, 20, 23] in Nigeria have shown that children in urban settings have lower mortality risks, Akinyemi et al. [22] reported no significant association between place of residence and under-five mortality in Nigeria.

Earlier epidemiologic studies have not concomitantly examined the factors influencing mortality at the different stages of early childhood development [4, 5] in a single analytical model. Given the varying roles of social determinants of health (SDH) across the three different stages of early childhood development, results from multinomial modeling will be more insightful to implementing targeted and universal child survival interventions. With respect to statistical methods, standard errors are higher by fitting separate dichotomous models of early childhood mortality (as used in the past studies), compared to a single multinomial model [24, 25]. Also, some of these studies in Nigeria have been hospital-based [26, 27] or limited to a specific geographical region, [26–31] making generalizability of findings somewhat challenging. Despite the high post-neonatal mortality rate in Nigeria (i.e., 31 deaths per 1000 live births) [32], only a handful of studies [17, 33] have been conducted. To fill these research gaps, we performed a secondary analysis of the latest (2016/2017) Multiple Indicator Cluster Survey (MICS) dataset [34]. By focusing on SDG-implementation era, this study extends the current evidence on social determinants of child survival in Nigeria, and other similar settings.

Nigeria needs to implement impactful policies/interventions towards making child-related SDG-3 targets, and the social inclusion perspective of SDG-10 targets (i.e., to reduce inequalities within and among countries) achievable by 2030. Interventions and policies that would drastically reduce childhood mortalities hinge on a sound understanding of the complex variations in the associations of the social determinants of health in the communities, and across the early child developmental stages. The objectives of this study, therefore, were to: (1) identify the social determinants of age-specific childhood (0–59 months) mortalities, which are disaggregated into neonatal mortality (0–27 days), post-neonatal mortality (1–11 months) and child mortality (12–59 months), and (2) estimate the within- and between-community variations of mortality among under-five children in Nigeria. In line with the expectations of the 2014 United Nations’ call for data revolution that would expedite sustainable development [35], this study provides evidence to guide stakeholders in planning for effective child survival strategies in the Nigerian communities during the Sustainable Development Goals era.

### Theoretical focus

In order to identify the key socio-economic variables influencing childhood mortality in Nigeria, we adapted the Mosley-Chen framework [36]. We hypothesized that micro-level/compositional factors (i.e., child, maternal, and household-level factors) and macro-level/contextual factors (i.e., community-level factors) influence childhood mortality risk across each of the successive developmental stages and that these relationships occur at hierarchical levels. Also, siblings are likely to share the same biological and social factors, especially those who are born within 5 years of each other as was the case in this study. However, children residing in different communities will likely have different mortality risks. We postulated that the mortality risks will vary across communities in Nigeria because of a complex mix of compositional and contextual factors influencing them. Multilevel analysis was employed to measure the extent by which compositional and contextual factors account for variation in mortality across the early child developmental stages at the community-level.

## Methods

### Study setting and population

The data for this study were drawn from the 2016/2017 MICS in Nigeria [34]. With a population of 200 million, Nigeria—a country located in West Africa, is the most populated country in sub-Saharan Africa. It has six geo-political regions (i.e., North-West (NW), North-East (NE), North-Central (NC), South-West (SW), South-East (SE), and South-South (SS), which are further divided into 36 states and Federal Capital Territory (FCT).

### Study design and data collection

The 2016/2017 MICS is the fifth-round of the national representative household survey, which was conducted between September 2016 and January 2017 in Nigeria [34]. The household survey is designed by the United Nations to provide national and subnational estimates of maternal and child indicators to monitor the SDG targets. Using multi-stage stratified cluster sampling technique in all 36 states and the FCT of Nigeria, the sample size was 36,176 women aged 15–49 years (out of which 34,376 women were interviewed, 95% response rate). The sampling frame which was within the states were stratified into urban (36.6%) and rural areas (63.4%). In total, 33,901 households from 2239 enumeration areas (i.e., primary sampling unit) were covered during fieldwork. Household is a unit consisting of family members and servants living together in a house, while community refers to the primary sampling unit (PSU), comprising of cluster of geographical and administratively distinct areas of homogenous households. The complete description of sample size calculation and sampling technique have been provided in the full 2016/2017 Nigeria MICS report [32]. For the purpose of this study, we merged birth history datafile with maternal and household files. To minimize recall bias, our inclusion criteria included every live birth delivered to all women aged 15–49 years in each household within 5 years prior to survey commencement (i.e., September 2011–September 2016). The children without documented survival outcome (i.e., alive, or dead), as well as the dates of birth and deaths were excluded from the analysis. Overall, a subpopulation of all 29,786 under-five children delivered to 18,497 women aged 15–49 years (irrespective of their marital status) was analyzed. These mothers were nested within 16,151 households and 2227 communities. The average number of children per community was 13.4.

#### Outcome variable

We considered under-five mortality as the omnibus outcome variable. This was generated from the variables that captured survival status, age at death, and current age of living children. The outcome variable was categorized into four levels, (i.e., alive (reference), and three age- and stage-specific mortality: neonatal, post-neonatal, and child mortality). Neonatal mortality is defined as under-five death occurring from birth to 27 days of life. Post-neonatal mortality is under-five death happening between 28 days and 11 months, and child mortality is under-five death occurring between 12 months and 59 months (i.e., toddler/pre-school stages).

#### Explanatory variables

The selection of independent variables was guided by data availability, theoretical focus of this study—Mosely-Chen framework [36], and evidence from literature that the potential covariables are expected to influence the outcome (child survival). The explanatory variables were layered across the child, maternal, household, and community-levels (Table 1). From the existing data, we generated variables on maternal media exposure (accessibility to newspaper/magazine or listening to the radio or watching television), housing condition index (using principal components analysis (PCA) of three variables—quality of roof, exterior wall and floor), access to drinking water, sanitation, indoor pollution, community level of maternal education, and community infrastructural development. Concerning marital status, the variable was defined as currently married/in union, formerly married/in union, and never married/in union. Also, maternal wealth index was used as a proxy for relative socioeconomic condition of women. In MICS, there is no information about a standard measure of absolute socioeconomic status (income). For this reason, we have used wealth index. The wealth index is a composite variable that was constructed using PCA of assets owned by mothers, such as television, car, bicycle, mobile phones etc. The wealth index was categorized into low (i.e., poorest and poor), middle, and high (i.e., rich and richest) socioeconomic status. The wealth index does not give information about current maternal income and expenditure. The community-level of maternal education was calculated as the proportion of households in a community that reported at least maternal secondary education. The scores were aggregated into three levels based on quartile classification (i.e., 2nd quartile = 50%, and 3rd quartile = 75%); (lower education < 50% of overall score), moderate education 50% ≤ x < 75% of the overall score, high education (75% ≤ x ≤ 100% of overall score). This study used proportion of households with access to electricity as a proxy for community infrastructural development. The variable has two groups based on median value. This variable was used primarily because of existing evidence that access to electricity strongly drives not only the development of social infrastructure, but also community health service delivery [37–41]. In Nigeria, electricity is mainly provided by the government, and where electricity is available, it is accessed by most of the population [42, 43]. Overall, missing data ranged from 0 to 33.2%. Except for access to antenatal care (ANC), frequency of ANC visits, skilled birth attendants during delivery, and institutional delivery, no variable had missing variable above 30%.

**Table 1:** Definition of variables

Variable	Description
**Dependent variable**	
Under-five survival status	Alive = 0, neonatal mortality = 1, post-neonatal mortality = 2, child mortality = 3
**Independent variables**	
**Child-level**	
Child’s sex	Male = 0, female = 1
Gestation type	Singleton = 0, multiple = 1
Birth order	First birth = 0, 2–3 = 1, 4–6 = 2, ≥7 = 3
Previous birth interval	< 2 years = 0, first birth = 1, ≥2 years = 2
Maternal age at birth	< 20 years = 0, 20–34 years = 1, ≥35 years = 2
**Maternal level**	
Maternal education	None/primary = 0, secondary/technical = 1, post-secondary = 2
Maternal wealth index	Poor = 0, middle = 1, rich = 2
Maternal media exposure	High = 0, medium = 1, low = 2, none = 3
Death of previous children at the time of the survey	< 3 = 0, 3–4 = 1, ≥5 = 2
Parity	< 3 = 0, 3–4 = 1, ≥5 = 2
Access to antenatal care (ANC)	None = 0, skilled = 1, unskilled = 2
Frequency of ANC visits	None = 0, 1–7 = 1, ≥8 = 2
Skilled birth attendants during delivery	None = 0, skilled birth attendants = 1, unskilled/friend = 2
Institutional delivery	Home = 0, health facility = 1
Marital status	Currently married/in union = 0, formerly married/in union = 1, never married/in union = 2
History of contraceptive use	Yes = 0, no = 1
Ever drank alcohol	Yes = 0, no = 1
Ever smoked cigarette	Yes = 0, no = 1
**Household-level**	
Sex of household head	Male = 0, female = 1
Household head education	None/primary = 0, secondary/technical = 1, post-secondary = 2
Ethnic group	Hausa = 0, Igbo = 1, Yoruba = 2, others = 3
Housing condition	Inadequate = 0, adequate = 1
Polygamy	Yes = 0, no = 1
Access to drinking water	Unimproved source = 0, improved source = 1
Sanitation	Unimproved = 0, improved = 1
Indoor pollution	Polluting fuel = 0, clean fuel = 1
Household size	Discrete
**Community-level**	
Place of residence	Urban = 0, rural = 1
Community infrastructural development (based on proportion of households with electricity in the community)	Low = 0, high = 1
Region	North-Central (NC) = 0, North-East (NE) = 1, North-West (NW) = 2, South-East (SE) = 3, South-South (SS) = 4, South-West (SW) = 5
Aggregated (community) level maternal education (based on proportion of households with women who had at least secondary education in the community)	Low = 0, medium = 1, high = 2

### Statistical analysis

All statistical analyses were performed using Stata™ software version 15.1 [44]. Parallel to MICS methodology of mortality estimation [32], neonatal and post-neonatal mortality rates were estimated using cumulative incidence (otherwise known as incidence proportion); and child and under-five mortality rates were derived from real sample cohorts using life tables. Given the hierarchical nature of the data and multinomial outcome, we performed multilevel multinomial logistic regression. By using survey analysis procedure (svyset cluster command in Stata™ software), bivariate analyses were conducted using Chi-square test for categorical variables and one-way analysis of variance (ANOVA) for discrete variable (household size) to select the potential covariates for multinomial regression models. To ensure representativeness of data, sample weights were applied. We used log-likelihood, Bayesian Information Criterion (BIC) and Akaike’s Information Criterion (AIC) to ascertain model goodness-of-fit. Missing data were addressed with listwise deletion.

#### Model building

##### Measures of association (fixed-effects)

The association between survival outcomes, and macro-level/contextual variables (community-level) and micro-level (child, maternal and household-level) variables were examined separately. Hence, six blocks of models were fitted, reflecting intercept-only, child, maternal, household, community-level factors, and parsimonious final model. In the interest of achieving a parsimonious model, we used a backward elimination strategy, retaining variables with *p-value* ≤ 0.25 from bivariate analyses as candidate variables in models 1–4. In the final model (model 5), variables with *p-value* < 0.05 from models 1–4 were selected. Based on the parsimonious approach, the covariates that were entered into the final multivariate model included child’s sex, gestation type, previous birth interval, maternal age at birth, maternal education, wealth index, death of previous children, parity, skilled birth attendants during delivery, contraceptive use, place of residence, and region. Exponentiating the coefficients (β) from multilevel logistic regression, we obtained the relative risk ratios (RRR) and their 95% confidence intervals (95%CI) [44]. We tested for interaction effects between the significant covariates in the multivariate models but found them not statistically significant; hence dropped from the analysis.

##### Measures of variation (random-effects)

Considering that small average sample sizes (≤2) at hierarchical levels could bias variance estimates and effect sizes (Type-I error) in multilevel analysis, we collapsed child, maternal and household-levels into a single level (i.e., child/maternal/household) [45]. The limited number of children at maternal-level (mean = 1.6) and household-level (mean = 1.8) could not allow for four-level model, with random effects at child, maternal, household and community-levels. Parallel to previous multilevel studies [46, 47], we had two levels of data hierarchies—child/maternal/household (micro) and community (macro) levels. With micro-and macro-levels defined as random effects, we examined whether and how much variation of child survival outcomes in the communities can be attributed to the compositional and contextual variables. The extent of the variability in mortality risks from community-to-community was estimated by median odds ratios (MOR) and percentage change in variance (PCV) [48]. MOR is preferable to intra-cluster correlation (ICC) because it quantifies the community-level variance (unexplained contextual heterogeneity) on the odds ratio scale, making it easier to interpret [48]. The MOR value is always equal or greater than one. MOR of 1 implies that no variation in mortality risk across the developmental stages exist between communities. MOR was computed using the following equation: [48]

1 $$ MOR={e}^{0.95\surd {V}_c}\kern1em $$

Where V_c_ is the community-level variance.

The proportional change in variance assesses the influence of compositional and contextual variables on inter-community variations of the outcome variable; we estimate this by comparing intercept-only model (model 0) with the five other models (models 1–5).

#### Predicted probabilities

Using estimates in the final model (model 5), we predicted the probabilities of death in each of neonatal, post-neonatal, and toddler/preschool stages by holding the final variables in the model constant at their mean values. Pairwise comparisons of predictive margins of significant correlates were also performed [44].

## Results

### Sample characteristics

Table 2 shows the socio-economic and demographic distribution of the study participants. Out of the weighted sample of 30,960 live births during the interview period, 29,729 (96.0%) were reported as singletons. The male to female ratio was 1.03:1. About 1 in 7 children were delivered by teenage mothers and 17.1% by women aged ≥35 years. More than half (66.6%) were children of women with no formal or primary education. In 44.5% of the children, their mothers were poor. More children were reported to be residing in NW region (39.1%), rural area (69.9%), and from infrastructurally less-developed communities (52.4%).

**Table 2:** Characteristics of study participants according to survival status, 2016/2017 MICS, Nigeria

Variable	Sample size Mean (SD)/ n (%)	Alive Mean (SD)/ n (%)	Neonatal mortality Mean (SD)/ n (%)	Post-neonatal mortality Mean (SD)/ n (%)	Child mortality Mean (SD)/ n (%)	*P*-value
**Child-level factors**						
Child’s sex (*N* = 30,959)						0.0009
Male	15,725 (50.8)	14,165 (50.4)	679 (57.9)	483 (54.4)	398 (49.0)	
Female	15,234 (49.2)	13,919 (49.6)	494 (42.1)	406 (45.6)	415 (51.0)	
Gestation type (*N* = 30,957)						< 0.001
Singleton	29,729 (96.0)	27,135 (96.6)	1003 (85.5)	820 (92.3)	771 (94.9)	
Multiple	1228 (4.0)	974 (3.4)	171 (14.5)	69 (7.7)	41 (5.1)	
Birth order (*N* = 30,960)						< 0.001
First	5436 (17.6)	4892 (17.4)	257 (21.9)	164 (18.5)	122 (15.0)	
2–3	10,358 (33.5)	9617 (34.2)	287 (24.5)	240 (27.0)	213 (26.2)	
4–6	9971 (32.2)	9108 (32.4)	309 (26.3)	278 (31.3)	276 (34.0)	
≥ 7	5195 (16.8)	4466 (15.9)	321 (27.3)	207 (23.2)	202 (24.8)	
Previous birth interval (*N* = 30,960)						< 0.001
< 2 years	6601 (21.3)	5630 (20.0)	413 (35.2)	292 (32.8)	266 (32.7)	
First birth	5501 (17.8)	4942 (17.6)	270 (23.0)	166 (18.7)	123 (15.2)	
≥ 2 years	18,858 (60.9)	17,512 (62.4)	491 (41.8)	431 (48.5)	423 (52.1)	
Maternal age at birth (*N* = 30,960)						< 0.001
< 20 years	4320 (14.0)	3781 (13.5)	241 (20.5)	153 (17.3)	144 (17.8)	
20–34 years	21,338 (68.9)	19,547 (69.6)	709 (60.4)	579 (65.1)	504 (62.0)	
≥ 35 years	5302(17.1)	4757 (16.9)	224 (19.1)	157 (17.6)	165 (20.3)	
**Maternal-level factors**						
Maternal education (*N* = 30,960)						< 0.001
None/primary	20,609 (66.6)	18,333 (65.3)	842 (71.7)	718 (80.8)	716 (88.0)	
Secondary	7981 (25.8)	7498 (26.7)	253 (21.5)	140 (15.7)	90 (11.1)	
Post-secondary	2369 (7.7)	2253 (8.0)	79 (6.7)	31 (3.47)	7 (0.9)	
Maternal wealth index (*N* = 30,960)						< 0.001
Poor	13,912 (44.9)	12,287 (43.7)	552 (47.1)	522 (58.7)	552 (67.9)	
Middle	6068 (19.6)	5471 (19.5)	260 (22.1)	176 (19.8)	161 (19.8)	
Rich	10,980 (35.5)	10,326 (36.8)	362 (30.8)	191 (21.5)	101 (12.4)	
Maternal media exposure (*N* = 30,956)						< 0.001
High	10,047 (32.5)	9296 (33.1)	367 (31.3)	227 (25.5)	158 (19.4)	
Medium	4972 (16.1)	4588 (16.3)	158 (13.5)	118 (13.2)	107 (13.2)	
Low	3542 (11.4)	3221 (11.5)	131 (11.2)	100 (11.2)	91 (11.1)	
None	12,394 (40.0)	10,976 (39.1)	517 (44.1)	444 (50.0)	457 (56.3)	
Death of previous children (*N* = 30,960)						< 0.001
< 3	28,584 (92.3)	26,557 (94.6)	834 (71.1)	640 (72.0)	553 (68.0)	
3–4	1882 (6.1)	1252 (4.5)	254 (21.7)	174 (19.6)	201 (24.7)	
≥ 5	494 (1.6)	275 (1.0)	85 (7.3)	75 (8.4)	60 (7.3)	
Parity (*N* = 30,960)						
< 3	7847 (25.3)	7293 (26.0)	262 (22.4)	168 (18.9)	123 (15.2)	< 0.001
3–4	10,136 (32.7)	9338 (33.3)	317 (27.0)	262 (29.5)	219 (27.0)	
≥ 5	12,977 (41.9)	11,453 (40.8)	594 (50.7)	459 (51.6)	470 (57.9)	
Access to ANC (*N* = 20,775)^b^						< 0.001
None	6726 (32.4)	5972 (31.6)	267 (33.2)	266 (45.0)	221 (44.9)	
Skilled	12,600 (60.7)	11,667 (61.8)	457 (56.8)	257 (43.5)	220 (44.6)	
Unskilled	1449 (7.0)	1250 (6.6)	80 (9.9)	68 (11.5)	51 (10.4)	
Freq of ANC visits (*N* = 20,775)^b^						< 0.001
None	6726 (32.4)	5972 (31.6)	267 (33.2)	266 (45.0)	221 (44.9)	
1–7	10,416 (50.1)	9541 (50.5)	381 (47.4)	265 (44.9)	228 (46.4)	
≥ 8	3633 (17.5)	3375 (17.9)	155 (19.3)	59 (10.1)	43 (8.7)	
Skilled birth attendants during delivery (*N* = 20,786)^b^						< 0.001
None	2646(12.7)	2318 (12.3)	113 (14.0)	97 (16.3)	118 (24.0)	
Skilled	8072 (38.8)	7540 (39.9)	310 (38.4)	135 (22.7)	88 (17.8)	
Unskilled	10,068 (48.4)	9035 (47.8)	385 (47.7)	362 (61.0)	286 (58.2)	
Institutional delivery (*N* = 20,783)^b^						< 0.001
Home	13,358 (64.3)	11,971 (63.3)	516 (64.2)	457 (77.4)	415 (84.3)	
Health facilities	7425 (35.7)	6926 (36.7)	288 (35.8)	134 (22.6)	77 (15.7)	
Marital status (*N* = 30,934)						0.5392
Currently married/in union	29,748 (96.2)	26,998 (96.2)	1120 (95.7)	852 (96.3)	778 (95.7)	
Formerly married/in union	803 (2.6)	717 (2.6)	32 (2.8)	25 (2.9)	29 (3.6)	
Never married/in union	382 (1.2)	351 (1.3)	18 (1.6)	8 (0.9)	6 (0.7)	
Contraceptive use (*N* = 26,807)						< 0.001
Yes	1947 (7.3)	1832 (7.6)	55 (5.4)	46 (5.6)	15 (1.9)	
No	24,860 (92.7)	22,363 (92.4)	973 (94.6)	776 (94.4)	749 (98.1)	
Alcohol intake (*N* = 30,807)						< 0.001
Yes	3649 (11.8)	3401 (12.2)	124 (10.6)	81 (9.2)	42 (5.21)	
No	27,158 (88.2)	24,547 (87.8)	1045 (89.4)	801 (90.8)	765 (94.8)	
Smoking experience (*N* = 30,898)						0.02
Yes	213 (0.7)	180 (0.6)	13 (1.2)	14 (1.6)	5 (0.6)	
No	30,685 (99.3)	27,855 (99.4)	1153 (98.8)	869 (98.4)	808 (99.4)	
**Household-level factors**						
Sex of household head (*N* = 30,960)						0.502
Male	29,614 (95.7)	26,844 (95.6)	1132 (96.4)	851 (95.7)	787 (96.8)	
Female	1346 (4.4)	1240 (4.4)	42 (3.6)	38 (4.3)	26 (3.2)	
Household head education (*N* = 30,881)						< 0.001
None/primary	18,114 (58.7)	16,116 (57.5)	760 (65.1)	614 (69.2)	623 (77.1)	
Secondary	8320 (26.9)	7702 (27.5)	267 (22.9)	207 (23.3)	143 (17.7)	
Post-secondary	4448 (14.4)	4199 (15.0)	140 (12.0)	66 (7.5)	42 (5.2)	
Ethnic group (*N* = 30,960)						< 0.001
Hausa	17,484 (56.5)	15,505 (55.2)	714 (60.8)	611 (68.7)	655 (80.6)	
Igbo	2409 (7.8)	2278 (8.1)	69 (5.9)	41 (4.6)	21 (2.2)	
Yoruba	2929 (9.5)	2755 (9.8)	104 (8.8)	46 (5.2)	21 (2.6)	
Others	8140 (26.3)	7546 (26.9)	287 (24.4)	191 (21.5)	116 (14.2)	
Housing condition (*N* = 30,960)						< 0.001
Inadequate	13,172(42.5)	11,694 (41.6)	511 (43.5)	480 (54.1)	487 (59.9)	
Adequate	17,788 (57.5)	16,391 (58.4)	663 (56.5)	408 (45.9)	326 (40.1)	
Polygamy (*N* = 30,960)						< 0.001
Yes	10,880 (35.1)	9699 (34.5)	432 (36.9)	375 (42.2)	373 (45.9)	
No	20,079 (64.9)	18,385 (65.5)	741 (63.1)	514 (57.8)	439 (54.1)	
Household access to drinking water (N = 30,959)						< 0.001
Unimproved	10,820 (35.0)	9587 (34.1)	480 (40.9)	405 (45.6)	348 (42.8)	
Improved	20,139 (65.1)	18,497 (65.9)	694 (59.1)	484 (54.4)	465 (57.2)	
Household sanitation (*N* = 30,959)						< 0.001
Unimproved	15,926 (51.4)	14,266 (50.8)	619 (52.7)	548 (61.6)	493 (60.7)	
Improved	15,033 (48.6)	13,817 (49.2)	555 (47.3)	341 (38.4)	320 (39.3)	
Indoor pollution (*N* = 30,960)						< 0.001
Polluting fuel	29,120 (94.1)	26,331 (93.8)	1123 (95.7)	860 (96.7)	806 (99.1)	
Clean fuel	1840 (5.9)	1753 (6.2)	50 (4.3)	29 (3.3)	7 (0.9)	
Household size (*N* = 30,960)						< 0.001
Mean (standard deviation)^a^	7.8 (4.1)	7.8 (4.1)	7.2 (4.1)	7.2 (3.8)	7.6 (3.9)	
**Community-level factors**						
Place of residence (*N* = 30,960)						< 0.001
Urban	9327 (30.1)	8710 (31.0)	323 (27.5)	162 (18.2)	131 (16.2)	
Rural	21,633 (69.9)	19,374 (69.0)	850 (72.5)	727 (81.8)	681 (83.8)	
Region (*N* = 30,960)						
North-Central (NC)	5084 (16.4)	4636 (16.5)	216 (18.4)	136 (15.3)	96 (11.8)	< 0.001
North-East (NE)	6517 (21.1)	5950 (21.2)	213 (18.1)	173 (19.4)	181 (22.2)	
North-West (NW)	12,113 (39.1)	10,651 (37.9)	534 (45.5)	453 (50.9)	475 (58.5)	
South-East (SE)	1604 (5.2)	1513 (5.4)	42 (3.6)	33 (3.7)	15 (1.9)	
South-South (SS)	2370 (7.7)	2252 (8.0)	52 (4.4)	45 (5.0)	21 (2.6)	
South-West (SW)	3272 (10.6)	3081 (11.0)	117 (9.9)	49 (5.6)	25 (3.0)	
Infrastructural development (*N* = 30,960)						< 0.001
Low	16,235 (52.4)	14,452 (51.5)	657 (56.0)	555 (62.5)	571 (70.2)	
High	14,724 (47.6)	13,632 (48.5)	516 (44.0)	334 (37.5)	242 (29.8)	
Comm. Maternal education (*N* = 30,960)						< 0.001
Low	16,451 (53.1)	14,434 (51.4)	727 (62.0)	627 (70.5)	663 (81.6)	
Medium	5842 (18.9)	5466 (19.5)	183 (15.6)	116 (13.1)	76 (9.4)	
High	8666 (28.0)	8184 (29.1)	263 (22.4)	146 (16.4)	74 (9.0)	

^a^One-way ANOVA, ^b^data available for only women with a live birth in the 2 years preceding the survey

### Differentials of age-specific mortality rates

Of the weighted 30,960 live births included in this study, 2840 died before reaching 5 years—121.6 deaths per 1000 live births, 95%CI: 116.8–126.5. The NMR was 37.9 deaths per 1000 live births, 95%CI: 34.9–41.1, post-neonatal mortality rate was 28.7 deaths per 1000 live births, 95%CI: 26.2–31.4, and child mortality rate was 54.3 deaths per 1000 children surviving to age one, 95%CI: 50.2–58.7. Among the children who died, 41.3% (95%CI: 38.8–43.9%) occurred at neonatal stage, 30.1% (95%CI: 28.0–32.3%) at post-neonatal stage, and 28.6% (95%CI: 26.2–31.1%) at toddler/preschool stage. More male children died during the neonatal (57.9%) and post-neonatal (54.4%) stages, but more female deaths (51%) occurred during toddler/preschool stage (*p-value* < 0.001) (Table 2). Uniformly across the early child developmental stages, deaths were significantly lower among women who were rich, in monogamous settings, and resided in houses deemed of adequate quality. In all the stages, mortality was higher in children whose mother had many children (grand multiparity), lived in rural communities, communities with low level of maternal education, less infrastructurally developed communities, and lived in NW region of Nigeria (Additional file 1: Table S1).

### Measures of variation (random effects)

In the intercept-only model (model 0), the estimated community-level variance was 0.49 (95%CI: 0.41–0.60), implying a significant variation of mortality risks across the communities. The heterogeneity at the community-level was confirmed by MOR of 1.95. As shown in Tables 3, 4, 5 and 6, the variations in under-five mortalities across the communities were mostly explained by maternal-level variables (PCV = 38.8%), followed by community-level variables (PCV = 28.6%), and child-level variables contributed the least (PCV = 14.3%). The combined effect of child, maternal, household and community-level variables (model 5) did not further reduce variability in the risk of under-five mortality between communities, compared to model that included only maternal-level variables (model 2). Also, in both model 2 (maternal-level variables) and model 5 (compositional and contextual variables), the community heterogeneity decreased from 1.95 to 1.68, thus, there is evidence to suggest residual variability of childhood mortality risks at the community-level.

**Table 3:** Multivariate multilevel multinomial regression of child-level factors associated with age-specific under-five mortality, 2016/217 Nigeria MICS

	Model 0	Model 1 (child-level factors) Ref: alive (***N*** = 30,957)		
	Intercept-only RRR (95%CI)	Neonatal mortality (0–27 days) RRR (95%CI)	Post-neonatal mortality (28 days-11 months) RRR (95%CI)	Child mortality (12 months to 11 months) RRR (95%CI)
**Fixed effects**				
Child’s sex				
Male		Ref.	Ref.	Ref.
Female		0.72 (0.61–0.85)***	0.84 (0.71–0.98)*	1.04 (0.85–1.27)
Gestation type				
Singleton		Ref.	Ref.	Ref.
Multiple		5.64 (4.25–7.49)***	2.76 (2.03–3.75)***	1.68 (1.09–2.58)*
Birth order				
First		Ref.	Ref.	Ref.
2–3		1.14 (0.58–2.25)	0.47 (0.10–2.17)	0.67 (0.13–3.49)
4–6		1.38 (0.68–2.80)	0.59 (0.13–2.74)	1.02 (0.19–5.43)
≥ 7		2.83 (1.37–5.84)**	0.88 (0.19–4.13)	1.44 (0.26–7.88)
Previous birth interval				
< 2 years		Ref.	Ref.	Ref.
First birth		0.99 (0.49–2.00)	0.35 (0.08–1.66)	0.41 (0.08–2.10)
≥ 2 years		0.37 (0.31–0.44)***	0.47 (0.39–0.57)***	0.52 (0.43–0.63)***
Maternal age at birth				
< 20 years		Ref.	Ref.	Ref.
20–34 years		0.59 (0.45–0.76)***	0.71(0.54–0.93)*	0.54 (0.40–0.71)***
≥ 35 years		0.55 (0.39–0.78)***	0.64 (0.44–0.95)*	0.55 (0.38–0.80)**
**Random effects (community-level)**				
Variance (SE)	0.49 (0.05)	0.42 (0.05)		
95%CI	0.41,0.60	0.34,0.53		
PCV	Ref.	14.3%		
MOR	1.95	1.85		

***p-value< 0.001 **p-value< 0.01 *p-value< 0.05; *SE* Robust standard error, *RRR* Relative risk ratio, *CI* Confidence interval, *PCV* Proportional change in variance, *MOR* Median odds ratio

**Table 4:** Multivariate multilevel multinomial regression of maternal-level factors associated with age-specific under-five mortality, 2016/2017 Nigeria MICS

	Model 0	Model 2 (maternal-level factors) Ref: alive (***N*** = 18,120)		
	Intercept-only RRR (95%CI)	Neonatal mortality (0–27 days) RRR (95%CI)	Post-neonatal mortality (28 days-11 months) RRR (95%CI)	Child mortality (12 months to 11 months) RRR (95%CI)
**Fixed effects**				
Maternal education				
None/primary		Ref.	Ref.	Ref.
Secondary		0.72 (0.53–0.99)*	0.69 (0.48–0.99)*	0.62 (0.41–0.93)*
Post-secondary		0.92 (0.51–1.65)	0.74 (0.39–1.41)	0.28 (0.09–0.82)*
Maternal wealth index				
Poor		Ref.	Ref.	Ref.
Middle		1.31 (0.99–1.73)	1.09 (0.80–1.49)	0.97 (0.71–1.33)
Rich		1.19 (0.87–1.62)	1.14 (0.79–1.65)	0.46 (0.31–0.69)***
Death of previous children				
< 3		Ref.	Ref.	Ref.
3–4		9.40 (6.66–13.27)***	5.37 (3.83–7.55)***	6.34 (4.55–8.83)***
≥ 5		10.00 (5.98–16.73)***	10.70 (6.27–18.25)***	7.75 (4.75–12.63)***
Parity				
< 3		Ref.	Ref.	Ref.
3–4		0.86 (0.67–1.10)	1.23 (0.90–1.67)	1.71 (1.19–2.47)**
≥ 5		0.57 (0.43–0.76)***	0.76 (0.54–1.06)	1.25 (0.86–1.82)
Freq of ANC visits^a^				
None		Ref.	Ref.	Ref.
1–7		1.04 (0.82–1.32)	0.92 (0.71–1.18)	1.19 (0.92–1.52)
≥ 8		1.26 (0.82–1.93)	0.65 (0.41–1.03)	1.19 (0.75–1.87)
Skilled birth attendants during delivery^a^				
None		Ref.	Ref.	Ref.
Skilled		1.22 (0.87–1.71)	0.73 (0.50–1.06)	0.58 (0.38–0.87)*
Unskilled		1.05 (0.79–1.40)	1.06 (0.78–1.45)	0.79 (0.60–1.04)
Contraceptive use				
Yes		Ref.	Ref.	Ref.
No		1.36 (0.91–2.03)	0.97 (0.59–1.60)	3.69 (1.40–9.75)*
**Random effects (community-level)**				
Variance (SE)	0.49 (0.05)	0.30 (0.05)		
95%CI	0.41,0.60	0.21,0.41		
PCV	Ref.	38.8%		
MOR	1.95	1.68		

***p-value< 0.001 **p-value< 0.01 *p-value< 0.05; ^a^data available for only women with a live birth in the 2 years preceding the survey; *SE* Robust standard error, *RRR* Relative risk ratio, *CI* Confidence interval, *PCV* Proportional change in variance, *MOR* Median odds ratio

**Table 5:** Multivariate multilevel multinomial regression of household-level factors associated with age-specific under-five mortality, 2016/2017 Nigeria MICS

	Model 0	Model 3 (household-level factors) Ref: alive (***N*** = 30,958)		
	Intercept-only RRR (95%CI)	Neonatal mortality (0–27 days) RRR (95%CI)	Post-neonatal mortality (28 days-11 months) RRR (95%CI)	Child mortality (12 months to 11 months) RRR (95%CI)
**Fixed effects**				
Housing condition				
Inadequate		Ref.	Ref.	Ref.
Adequate		1.14 (0.93–1.38)	0.82 (0.67–1.01)	0.62 (0.50–0.78)***
Polygamy				
Yes		Ref.	Ref.	Ref.
No		1.01 (0.85–1.197)	0.87 (0.73–1.04)	0.78 (0.63–0.95)*
Household access to drinking water				
Unimproved		Ref.	Ref.	Ref.
Improved		0.77 (0.62–0.95)*	0.76 (0.63–0.92)**	0.93 (0.75–1.14)
Household sanitation				
Unimproved		Ref.	Ref.	Ref.
Improved		1.02 (0.85–1.22)	0.80 (0.64–0.99)*	0.91 (0.75–1.11)
Indoor pollution				
Polluting fuel		Ref.	Ref.	Ref.
Clean fuel		0.78 (0.54–1.12)	0.79 (0.51–1.24)	0.20 (0.08–0.50)**
**Random effects (community-level)**				
Variance (SE)	0.49 (0.05)	0.41 (0.05)		
95%CI	0.41,0.60	0.34, 0.51		
PCV	Ref.	16.3%		
MOR	1.95	1.84		

***p-value< 0.001 **p-value< 0.01 *p-value< 0.05; *SE* Robust standard error, *RRR* Relative risk ratio, *CI* Confidence interval, *PCV* Proportional change in variance, *MOR* Median odds ratio

**Table 6:** Multivariate multilevel multinomial regression of community-level factors associated with age-specific under-five mortality, 2016/2017 Nigeria MICS

	Model 0	Model 4 (Community-level factors) Ref: alive (***N*** = 30,960)		
	Intercept-only RRR (95%CI)	Neonatal mortality (0–27 days) RRR (95%CI)	Post-neonatal mortality (28 days-11 months) RRR (95%CI)	Child mortality (12 months to 11 months) RRR (95%CI)
**Fixed effects**				
Place of residence				
Urban		Ref.	Ref.	Ref.
Rural		1.04 (0.81–1.33)	1.48 (1.12–1.95)**	1.25 (0.84–1.88)
Region				
North-Central (NC)		Ref.	Ref.	Ref.
North-East (NE)		0.80 (0.59–1.08)	1.0 (0.73–1.36)	1.38 (0.95–2.00)
North-West (NW)		1.08 (0.86–1.35)	1.36 (1.07–1.73)*	1.83 (1.32–2.53)***
South-East (SE)		0.75 (0.52–1.07)	1.09 (0.71–1.67)	0.99 (0.57–1.72)
South-South (SS)		0.61 (0.43–0.87)**	0.98 (0.66–1.45)	0.92 (0.55–1.54)
South-West (SW)		1.02 (0.75–1.38)	0.92 (0.61–1.39)	0.79 (0.43–1.44)
Comm. Maternal education				
Low		Ref.	Ref.	Ref.
Medium		0.73 (0.55–0.97)*	0.62 (0.47–0.82)***	0.41 (0.28–0.60)***
High		0.71 (0.53–0.95)*	0.55 (0.41–0.75)***	0.31 (0.20–0.46)***
**Random effects (community-level)**				
Variance (SE)	0.49 (0.05)	0.35 (0.04)		
95%CI	0.41,0.60	0.28, 0.44		
PCV	Ref.	28.6%		
MOR	1.95	1.75		

***p-value< 0.001 **p-value< 0.01 *p-value< 0.05; *SE* Robust standard error, *RRR* Relative risk ratio, *CI* Confidence interval, *PCV* Proportional change in variance, *MOR* Median odds ratio

### Measures of association (fixed effects)

The results from the bivariate analyses indicate that all the variables were significantly associated with under-five mortality, except marital status and sex of household head (Table 2). As shown by adjusted relative risk ratios (aRRR) in the final model (Table 7), the risks of dying during neonatal (0.67, 95%CI: 0.55–0.81, *p-value* < 0.001) and post-neonatal stages (0.80, 95%CI: 0.65–0.98, *p-value* = 0.035) were reduced for female compared to male child, but no significant reduction in the later stage, from 1 to 5 years of age.

**Table 7:** Multivariate multilevel multinomial regression of child, maternal, household and community-level factors associated with age-specific under-five mortality, 2016/2017 Nigeria MICS

	Model 0	Model 5 (Final model) Ref: alive (***N*** = 18,219)		
	Intercept-only RRR (95%CI)	Neonatal mortality (0–27 days) RRR (95%CI)	Post-neonatal mortality (28 days-11 months) RRR (95%CI)	Child mortality (12 months to 11 months) RRR (95%CI)
**Child-level factors**				
Child’s sex				
Male		Ref.	Ref.	Ref.
Female		0.67 (0.55–0.81)***	0.80 (0.65–0.98)*	1.02 (0.80–1.30)
Gestation type				
Single		Ref.	Ref.	Ref.
Multiple		5.77 (4.07–8.17)***	2.28 (1.52–3.44)***	1.33 (0.70–2.53)
Previous birth interval				
< 2 years		Ref.	Ref.	Ref.
First birth		1.26 (0.84–1.90)	1.82 (1.18–2.82)**	1.68 (1.07–2.63)*
≥ 2 years		0.38 (0.30–0.49)***	0.57 (0.44–0.73)***	0.48 (0.36–0.64)***
Maternal age at birth				
< 20 years		Ref.	Ref.	Ref.
20–34 years		0.46 (0.33–0.62)***	0.84 (0.58–1.23)	0.66 (0.44–0.99)*
≥ 35 years		0.36 (0.24–0.55)***	0.71 (0.42–1.21)	0.52 (0.30–0.93)*
**Maternal-level factors**				
Maternal education				
None/primary		Ref.	Ref.	Ref.
Secondary		0.85 (0.62–1.17)	0.68 (0.47–0.97)*	0.77 (0.50–1.17)
Post-secondary		1.13 (0.64–2.02)	0.74 (0.39–1.43)	0.34 (0.12–1.03)
Maternal wealth index				
Poor		Ref.	Ref.	Ref.
Middle		1.30 (0.98–1.71)	1.15 (0.85–1.57)	1.07 (0.80–1.45)
Rich		1.35 (0.94–1.93)	1.37 (0.94–2.01)	0.57 (0.35–0.92)*
Death of previous children				
< 3		Ref.	Ref.	Ref.
3–4		9.07 (6.48–12.68)***	5.23 (3.71–7.37)***	6.12 (4.42–8.47)***
≥ 5		10.01 (5.75–17.44)***	10.52 (6.21–17.82)***	7.78 (4.74–12.77)***
Parity				
< 3		Ref.	Ref.	Ref.
3–4		1.77 (1.25–2.53)***	2.42 (1.57–3.73)***	3.87 (2.46–6.08)***
≥ 5		1.54 (0.96–2.46)	1.76 (1.06–2.93)*	3.66 (2.18–6.17)***
Skilled birth attendants during delivery^a^				
None		Ref.	Ref.	Ref.
Skilled		1.39 (1.02–1.90)*	0.72 (0.49–1.07)	0.75 (0.50–1.12)
Unskilled		1.08 (0.81–1.45)	1.07 (0.78–1.47)	0.87 (0.66–1.15)
Contraceptive use				
Yes		Ref.	Ref.	Ref.
No		1.22 (0.82–1.84)	0.97 (0.58–1.60)	3.34 (1.27–8.81)*
**Community-level factors**				
Place of residence				
Urban		Ref.	Ref.	Ref.
Rural		1.08 (0.79–1.47)	1.66 (1.13–2.45)**	1.15 (0.74–1.79)
Region				
North-Central (NC)		Ref.	Ref.	Ref.
North-East (NE)		0.75 (0.53–1.06)	0.93 (0.64–1.34)	1.05 (0.67–1.64)
North-West (NW)		1.07 (0.82–1.40)	1.15 (0.86–1.54)	1.63 (1.09–2.41)*
South-East (SE)		0.45 (0.27–0.76)**	0.94 (0.54–1.65)	0.85 (0.40–1.80)
South-South (SS)		0.50 (0.32–0.79)**	0.99 (0.61–1.60)	0.57 (0.29–1.13)
South-West (SW)		1.28 (0.87–1.88)	1.00 (0.58–1.70)	0.96 (0.43–2.17)
**Random effects (community-level)**				
Variance (SE) 95%CI	0.49 (0.05) 0.41,0.60	0.30 (0.05) 0.22, 0.42		
PCV	Ref.	38.78%		
MOR	1.95	1.68		

***p-value< 0.001 **p-value< 0.01 *p-value< 0.05; ^a^data available for only women with a live birth in the 2 years preceding the survey; *SE* Robust standard error, *RRR* Relative risk ratio, *CI* Confidence interval, *PCV* Proportional change in variance, *MOR* Median odds ratio

Children of mothers born two or more years apart, compared to those closer together (less than two-year gap) had decreased risk of neonatal mortality (aRRR: 0.38, 95%CI: 0.30–0.49, *p-value* < 0.001), post-neonatal mortality (aRRR: 0.57, 95%CI: 0.44–0.73, *p-value* < 0.001), and child mortality (aRRR: 0.48, 95%CI: 0.36–0.64, *p-value* < 0.001). On the contrary, first births had higher risks of post-neonatal mortality (aRRR: 1.82, 95%CI: 1.18–2.82, *p-value* = 0.007) and child mortality (aRRR: 1.68, 95%CI: 1.07–2.63, *p-value* = 0.024) than births less than 2 years after a previous birth.

Children delivered as part of multiple births were about 6 times (aRRR: 5.77, 95%CI: 4.07–8.17, *p-value* < 0.001) more likely to die during neonatal stage, and about twice (aRRR: 2.28, 95%CI: 1.52–3.44, *p-value* < 0.001) the mortality risk at post-neonatal stage, compared to singletons. There were reduced neonatal, post-neonatal, and child mortality risks for children of older women, compared to teen mothers (Table 7).

Compared to children whose mothers were uneducated or had primary education, those with secondary education had lesser post-neonatal mortality risk (aRRR: 0.68, 95%CI: 0.47–0.97, *p-value* = 0.035). Also, for children aged 1–5 years, there was reduced mortality risk among those whose mothers were rich (aRRR: 0.57, 95%CI: 0.35–0.92, *p-value* = 0.02). Women with no history of contraceptive use had increased mortality risk for toddlers/preschoolers (aRRR: 3.34, 95%CI: 1.27–8.81, *p-value* = 0.015). Death of previous children reduced survival of subsequent children across the three child developmental stages. The mortality risk increases as the number of deaths of previous children increases (dose-response relationship). Grand multiparous women (≥ 5 deliveries) were more likely to experience increased mortality for children in post-neonatal stage (aRR: 1.76, 95%CI: 1.06–2.93, *p-value* = 0.03) and toddler/preschool stage (aRRR: 3.66, 95%CI: 2.18–6.17, *p-value* < 0.001). Unexpectedly, risk of neonatal mortality was higher among women who experienced skilled birth delivery compared to women with none (aRRR: 1.39, 95%CI: 1.02–1.90, *p-value* = 0.039). However, there was no statistically significant association between post-neonatal and child mortality. Further sensitivity analyses based on pairwise deletion (inclusion of missing data) showed similar associations between skilled birth delivery and neonatal mortality risk (aRRR: 1.38, 95%CI: 1.02–1.87, *p-value* = 0.037). Pairwise deletion also showed that skilled birth delivery was not significantly associated with post-neonatal mortality (aRRR: 0.85, 95%CI: 0.59–1.23, *p-value* = 0.387), and child mortality (aRRR: 0.70, 95%CI: 0.48–1.04, *p*-value = 0.075).

Children residing in rural areas had 2-fold increase in post-neonatal mortality risk compared to those in the urban areas (aRRR: 1.66, 95%CI: 1.13–2.45, *p-value* = 0.01), however, no statistically significant association was observed for neonatal and child mortalities. Living in SS and SE regions had protective effect on neonatal survival, while residing in NE region increased the mortality risk during toddler/pre-school stage.

### Predicted probabilities

Given that the compositional and contextual variables are at mean values, the adjusted predicted probability of dying during the neonatal stage is 0.028 (95%CI: 0.024–0.031, *p-value* < 0.001), post-neonatal stage is 0.025 (95%CI: 0.022–0.028, *p-value* < 0.001), and toddler/pre-school stage is 0.015 (95%CI: 0.013–0.018, *p-value* < 0.001).

## Discussion

Using the recent datasets of the Multiple Indicator Cluster Survey for Nigeria, we identified the key social determinants of under-five mortality across the neonatal, post-neonatal, and toddler/preschool stages. At each stage of early childhood development, there are different factors relating to child survival, which require different interventions. Our results show that high variation (39%) of under-five mortality across the Nigerian communities is mainly accounted for by maternal-level factors such as maternal education, contraceptive use, maternal wealth, parity, death of previous children and quality of perinatal care. The inclusion of other compositional and contextual factors had no significant additional effect on the community variation of childhood mortality risks. To our surprise somewhat, attendance of skilled providers during delivery was associated (only) with an increased neonatal mortality risk. Female child and singleton had decreased mortality risk during neonatal and post-neonatal stages. Also, teenage mothers experienced increased neonatal and child mortality risks. The significant factors associated with reduced mortality risk at post-neonatal stage were maternal secondary education and urban residence. Maternal wealth and contraceptive use lowered mortality risks among toddlers/pre-schoolers. While living in South-East and South-South regions of Nigeria lowered neonatal mortality risk, residence of North-West region had increased child mortality risk.

Our analysis showed an overall under-five mortality rate of 122 deaths per 1000 live births, which is similar to a rate of 120 deaths per 1000 live births reported in the 2016/2017 MICS report [32]. Also, the neonatal, post-neonatal and child mortality rates in this study (i.e., 38 deaths per 1000 live births, 29 deaths per 1000 live births, and 54 deaths per 1000 children surviving to age one, respectively) are consistent with those reported by MICS team (i.e., 39 deaths per 1000 live births, 31 deaths per 1000 live births, and 54 deaths per 1000 children surviving to age one, respectively). As one would expect, our estimates are comparable with the rates reported in the MICS report for the socioeconomic differentials of age- and stage-specific childhood mortality rates.

Consistent with existing literature from resource-limited countries [17, 49], we found that the probability of under-five mortality was highest during neonatal stage, followed by post-neonatal stage, and lowest in the toddler/preschool stage. In terms of child’s sex, our study found higher mortality risks among male children during neonatal and post-neonatal stages, and higher mortality risk among female children during toddler/pre-school stage. These findings align with reports from previous studies [17, 49–51]. Many authors argued that female survival in the early childhood is physiologically related [17, 49–51]. In addition, male neonates have increased risks of infections, jaundice, birth complications, congenital malformations, and more importantly pre-term births. According to Lawn et al. [50], the high risk of male neonatal deaths could be explained, in part, by the physiological fact that males’ lungs and other vital organs develop less rapidly in-utero and within first week of life, making them more susceptible to respiratory infections compared to females. In the same vein, male pre-term children are more likely to have placental problems and maternal history of hypertension (including pre-eclampsia) during their pregnancies, which might increase their risk of dying during neonatal and post-natal stages. By contrast, male-preference, inadequate care, and malnutrition among females are plausible reasons for the reversal in mortality risk among toddler/pre-schoolers [49].

Our study also highlights that mothers with secondary education had lesser risk of post-neonatal deaths, and wealthy mothers had lesser risk of child mortality. This observation could be due to better health literacy and access to financial resources. Strikingly, we did not observe statistically significant association between post-secondary education and mortality risk across the early childhood developmental stages. Furthermore, this study explicates the need for improving perinatal care in Nigeria because of its short and long-term benefits, especially during neonatal stage. However, we speculate that the observed association between skilled birth attendants during delivery and neonatal outcomes might be due to late presentation of expectant mothers at health facilities, when complications might have occurred and/or progressed. Late arrival for skilled birth deliveries could be the result of poor health seeking behaviour or long distance to health facilities. Given the complications arising from childbirth, such newborns may die shortly after delivery. This finding may also be indicative of sub-optimal quality of perinatal services in Nigeria. More needs to be done to support assisted deliveries by skilled health professional, which is currently low in Nigeria (43%) [52]. Past studies have suggested some of the contributing factors for poor performance of healthcare workers in the resource-limited countries [53–55]. These include burn-out due to shortage of skilled birth attendants, lack of motivation, inadequate supervision, weak patient-healthcare worker relationships, bad transportation system, and frequent stock-out of essential drugs [53–55]. With 18.3 skilled birth attendants per 10,000 population [56], Nigeria still struggles to achieve the recommended United Nations’ threshold of 44.5 skilled birth attendants per 10,000 population needed to achieve the maternal and child health targets of SDG-3 [57]. Also, there are other potential explanations for the association between skilled birth delivery and neonatal mortality risk. There is a possibility of under-reporting under-five mortalities among mothers with no or unskilled birth deliveries at the community level. Alternatively, it could indicate a functioning referral system that sends the high-risk deliveries to health facilities to a greater extent. It is also important to note that the unique relationship could be due to misclassification of cadre of skilled health workers by uneducated mothers. Another explanation might be iatrogenic injuries or nosocomial infections, or poor quality of care overall that is posing a risk of children dying.

After controlling for other variables, our findings suggest that rural residence influences mortality in post-neonates, unlike neonates and toddlers/pre-schoolers. Past studies [17, 19] in Nigeria have also reported that urban residence is a protective factor for post-natal and child mortality due to potential access to quality healthcare services. Our study further revealed that previous child death remained the strongest determinant of subsequent deaths among children (as determined with the highest relative risk ratios). In line with a study in rural Nepal, Gubhaju [58] asserts that death of previous children could lead to increased parity and shorter birth intervals, arising from early resumption of ovulation and unprotected coital exposure. However, from this study, short birth intervals have independent effect on mortality risks across the three developmental stages. Much attention has been given to the effects of birth spacing on child survival, however, the results are conflicting. Some authors argue that short and prolonged interpregnancy intervals are associated with increased risks of adverse perinatal outcomes [59–61]. Furthermore, this study identifies increased mortality risks among children of mothers born less than 2 years apart, part of multiple births, and had ≥ five deliveries. Some authors [61, 62] have opined that the detrimental effects of short previous birth intervals on childhood mortality might be due to biological (“maternal depletion syndrome”) and behavioural (competition of household resources among closely spaced siblings). Our analysis found evidence that the adverse influence of multiple births was highest for neonates and waned across the developmental stages. Taken with the findings from Bangladesh [51], the dwindling mortality risk for children who are part of multiple births might be an indication of additional supports given to mothers by the extended family members as the children grow. Also, it has been speculated that increased mortality risk among children delivered as part of multiple gestation during neonatal and post-neonatal stages could be partly due to prematurity, twin-to-twin transfusion syndrome [63], and vulnerability to infection due to low birth weight [64].

The limitations of the present study include a tendency for information bias arising from maternal self-reporting of data. There might be some recall bias/poor memory leading to (over)under-reporting of childhood mortality—heaping of deaths (i.e., displacement of age at death and preference for selecting certain ages/digits over others as interviewers fail to probe the exact timing of deaths because of its cultural implications). However, recall bias is unlikely to have played a major role since we included only the live births preceding 5 years to the survey. Due to absence of some variables in the dataset, the data did not allow us to adjust for child nutrition, prematurity, HIV, immunization, and birth weight. However, we used maternal wealth index in-lieu of food (in)security because of its association [65]. Also, cautious interpretations are needed because of the cross-sectional nature of this study, which do not allow for causal inference. Nevertheless, we have reported associations between social determinants of health and age-specific under-five mortality in Nigeria. As far as we know, no previous published research has investigated the factors associated with neonatal, post-neonatal, and child mortality for Nigeria, using multilevel multinomial logistic regression. By simultaneously fitting with multinomial regression models, standard errors were reduced, unlike separate binary logistic regression used in past studies. Our results were further strengthened by using an analytical approach on a national representative dataset, which allows for the findings to be generalized across Nigeria and other settings with similar characteristics. Further studies are needed to establish the disappearance of urban advantage during the neonatal and toddler/pre-school stages. Also, future investigations are necessary to validate the association between quality of perinatal care and childhood mortality, and mechanisms that connect maternal-level factors with community variation of childhood mortalities in Nigeria.

In conclusion, this study provides evidence to guide policymakers in Nigeria on the appropriate interventions to make child-health related Sustainable Development Goals achievable by 2030.

The study reinforces the importance of maternal-level factors in reducing childhood mortality. As maternal-level factors majorly contributed to community variation of under-five mortality in Nigeria, the government with other stakeholders should prioritize making education accessible for girls and in reducing economic hardships for women. In addition, focusing on other social factors operating at different stages of early childhood development, such as short spacing in-between pregnancies, teenage pregnancy, contraceptive use and urbanization, offer potentials of improving child survival in Nigeria. Also, community awareness and health counseling at the health facilities should include child spacing and contraceptive use. At the health facilities, healthcare workers should be alert to the potential mortality risks among high-risk children (especially those delivered to mothers with previous history of child loss), and are advised to take detailed history to screen newborns delivered to such high-risk mothers.

## Supplementary information

**Additional file 1: Table S1.** Differentials of age-specific childhood mortality rates, 2016/2017 MICS, Nigeria.

## Data Availability

Dataset for this study is open and publicly available at the official UNICEF MICS website [34].

## Abbreviations

AIC: Akaike’s Information Criterion
ANOVA: Analysis of variance
BIC: Bayesian Information Criterion
CI: Confidence Interval
FCT: Federal Capital Territory
ICC: Intraclass Correlation Coefficient
MDG: Millennium Development Goal
MICS: Multiple Indicator Cluster Survey
MOR: Median Odds Ratio
NMR: Neonatal Mortality Rate
PSU: Primary Sampling Unit
RRR: Relative risk ratio
SDG: Sustainable Development Goal
SE: Standard Error
U5MR: Under-five Mortality Rate

## References

[1] UNICEF (2018). Levels and trends in child mortality.

[2] World Health Organization (2015). Health in 2015: from MDGs, millennium development goals to SDGs, sustainable development goals.

[3] United Nations Economic and Social Council (2018). Progress towards the sustainable development goals.

[4] Abajobir AA, Abate KH, Abbafati C, Abbas KM, Abd-Allah F, GBD 2016 Mortality Collaborators H (2017). Global, regional, and national under-5 mortality, adult mortality, age-specific mortality, and life expectancy, 1970–2016: a systematic analysis for the global burden of disease study 2016. Lancet.

[5] Braveman P, Gottlieb L (2014). The social determinants of health: it’s time to consider the causes of the causes. Public Health Rep.

[6] Adewuyi EO, Zhao Y, Lamichhane R (2017). Risk factors for infant mortality in rural and urban Nigeria: evidence from the national household survey. Scand J Public Health.

[7] Antai D, Ghilagaber G, Wedrén S, Macassa G, Moradi T (2009). Inequities in under-five mortality in Nigeria: differentials by religious affiliation of the mother. J Relig Health.

[8] Adetoro GW, Amoo EO (2014). A statistical analysis of child mortality : evidence from Nigeria. J Demogr Soc Stat.

[9] Chukwu A, Okonkwo U (2015). Determinants of under-five mortality in Nigeria: an application of cox proportional hazard and cox frailty models. IOSR J Math.

[10] Wegbom AI, Wokoma DSA, Nnoka LC, Onyesom C (2016). What explains the high rate of infant mortality in rural Nigeria: biodemographic or socioeconomic factors?. Int J Heal Sci Res.

[11] Adebowale SA, Morakinyo OM, Ana GR (2017). Housing materials as predictors of under-five mortality in Nigeria: evidence from 2013 demographic and health survey. BMC Pediatr.

[12] Blackstone SR, Nwaozuru U, Iwelunmor J (2017). An examination of the maternal social determinants influencing under-5 mortality in Nigeria: evidence from the 2013 Nigeria demographic health survey. Glob Public Health.

[13] Morakinyo OM, Fagbamigbe AF (2017). Neonatal, infant and under-five mortalities in Nigeria: an examination of trends and drivers (2003-2013). PLoS One.

[14] Antai D (2011). Regional inequalities in under-5 mortality in Nigeria: a population-based analysis of individual- and community-level determinants. Popul Health Metrics.

[15] Adeolu M, Akpa O, Adeolu A, Aladeniyi I (2016). Environmental and socioeconomic determinants of child mortality: evidence from the 2013 Nigerian demographic health survey. Am J Public Heal Res.

[16] Akinyemi JO, Bamgboye EA, Ayeni O (2015). Trends in neonatal mortality in Nigeria and effects of bio-demographic and maternal characteristics. BMC Pediatr.

[17] Ezeh KO, Agho EK, Dibley JM, Hall JJ, Page NA (2015). Risk factors for postneonatal, infant, child and under-5 mortality in Nigeria: a pooled cross-sectional analysis. BMJ Open.

[18] Biradar R, Patel KK, Prasad JB (2019). Effect of birth interval and wealth on under-5 child mortality in Nigeria. Clin Epidemiol Glob Heal.

[19] Antai D, Moradi T (2010). Urban area disadvantage and under-5 mortality in Nigeria: the effect of rapid urbanization. Environ Health Perspect.

[20] Kayode GA, Adekanmbi VT, Uthman OA (2012). Risk factors and a predictive model for under-five mortality in Nigeria: evidence from Nigeria demographic and health survey. BMC Pregnancy Childbirth.

[21] Yaya S, Ekholuenetale M, Tudeme G, Vaibhav S, Bishwajit G, Kadio B (2017). Prevalence and determinants of childhood mortality in Nigeria. BMC Public Health.

[22] Akinyemi JO, Bamgboye EA, Ayeni O (2013). New trends in under-five mortality determinants and their effects on child survival in Nigeria: a review of childhood mortality data from 1990-2008. Afr Popul Stud.

[23] Izugbara C (2014). Whose child is dying? Household characteristics and under-five mortality in Nigeria. South African J Child Heal.

[24] Beyene J, Neupane B, McDonald S (2014). Identifying determinants and estimating the risk of inadequate and excess gestational weight gain using a multinomial logistic regression model. Open Access Med Stat.

[25] Lin Y, Deng X, Li X, Ma E (2014). Comparison of multinomial logistic regression and logistic regression: which is more efficient in allocating land use?. Front Earth Sci.

[26] Ibeneme C, Ezuruike E, Korie F, Chukwudi N (2019). Under-five mortality at the children’s emergency room of Federal Medical Centre, Umuahia, southeastern Nigeria. Int J Med Heal Dev.

[27] Azuh DE, Chinedu S, Samuel OW, Azuh A, Joshua G, Amoo EO (2019). Factors influencing the survival of under-five children among women visiting government health care facility in semi-urban communities in Nigeria. Cogent Arts Humanit.

[28] Akinwande MO, Ibrahim A, Ibrahim AA (2016). Analyzing infant and child (under-five) mortality in Zaria: a regression analysis approach. African Res Rev.

[29] Guerrier G (2013). Oluyide, Keramarou, Grais R. high maternal and neonatal mortality rates in northern Nigeria: an 8-month observational study. Int J Women's Health.

[30] Pen O (2013). Determinants of under–five mortality in South–Eastern Nigeria. MOJ Public Heal.

[31] Abu IN, Madu IA, Ajaero CK (2015). The prevalence and determinants of under-five mortality in Benue state, Nigeria. SAGE Open.

[32] UNICEF, NBS (2017). Multiple indicator cluster survey 2016–17, survey findings report. Abuja, Nigeria.

[33] Ekanem EE (1990). Causes of neonatal and postneonatal mortality in Nigerian children. Early Child Dev Care.

[34] UNICEF MICS (2018). Datasets.

[35] United Nations Data Revolution Group (2014). A world that counts: mobilizing the data revolution for sustainable development.

[36] Mosley W, Chen L (1984). An analytical framework for the study of child survival in developing countries. Popul Dev Rev.

[37] Opoku R, Adjei EA, Obeng GY, Severi L, Bawa A-R (2020). Electricity access, community healthcare service delivery, and rural development nexus: analysis of 3 solar electrified CHPS in off-grid communities in Ghana. J Energy.

[38] Cook P (2013). Rural electrification and rural development. Green Energy Technol.

[39] Vernet A, Khayesi JNO, George V, George G, Bahaj AS (2019). How does energy matter? Rural electrification, entrepreneurship, and community development in Kenya. Energy Policy.

[40] Riva F, Ahlborg H, Hartvigsson E, Pachauri S, Colombo E (2018). Electricity access and rural development: review of complex socio-economic dynamics and causal diagrams for more appropriate energy modelling. Energy Sustain Dev.

[41] Chen YJ, Chindarkar N, Xiao Y (2019). Effect of reliable electricity on health facilities, health information, and child and maternal health services utilization: evidence from rural Gujarat, India. J Health Popul Nutr.

[42] SEforALL Africa Hub (2020). Nigeria - sustainable energy for all.

[43] PWC Nigeria (2017). To achieve universal access to electricity, current approach to off-grid electricity needs to be changed.

[44] Stata version 15.1. 2017 [cited 2019 May 30]. Available from: https://www.stata.com/order/.

[45] Clarke P (2008). When can group level clustering be ignored? Multilevel models versus single-level models with sparse data. J Epidemiol Community Health.

[46] Uthman OA (2009). A multilevel analysis of individual and community effect on chronic childhood malnutrition in rural Nigeria. J Trop Pediatr.

[47] Sissoko D, Trottier H, Malvy D, Johri M (2014). The influence of compositional and contextual factors on non-receipt of basic vaccines among children of 12-23-month old in India: a multilevel analysis. PLoS One.

[48] Larsen K, Merlo J (2005). Appropriate assessment of neighborhood effects on individual health: integrating random and fixed effects in multilevel logistic regression. Am J Epidemiol.

[49] Naline G, Brinda V (2017). Predictors of age-specific childhood mortality in India.

[50] Lawn JE, Blencowe H, Darmstadt GL, Bhutta ZA (2013). Beyond newborn survival: the world you are born into determines your risk of disability-free survival. Pediatr Res.

[51] Bhowmik KR, Islam S (2016). Logistic regression and multiple classification analyses to explore risk factors of under-5 mortality in Bangladesh. Proceedings of the Pakistan Academy of Sciences: Pakistan Academy of Sciences B life and environmental sciences.

[52] National Population Commission (NPC) [Nigeria] and ICF (2019). Nigeria demographic and health survey 2018 key indicators report.

[53] Sumankuuro J, Crockett J, Wang S (2018). Perceived barriers to maternal and newborn health services delivery: a qualitative study of health workers and community members in low and middle-income settings. BMJ Open.

[54] Oguntunde O, Surajo IM, Dauda DS, Salihu A, Anas-Kolo S, Sinai I (2018). Overcoming barriers to access and utilization of maternal, newborn and child health services in northern Nigeria: an evaluation of facility health committees. BMC Health Serv Res.

[55] Yaya S, Bishwajit G, Uthman OA, Amouzou A (2018). Why some women fail to give birth at health facilities: a comparative study between Ethiopia and Nigeria. PLoS One.

[56] WHO (2017). World health statistics: monitoring health for the sustainable development goals.

[57] WHO (2016). Global strategy on human resources for health: workforce 2030 draft 1.0 submitted to the executive board (138th session).

[58] Gubhaju BB (1985). The effect of previous child death on infant and child mortality in rural Nepal. Stud Fam Plan.

[59] Kwarteng AG, Eyram AY (2017). Determinants of under-five mortality in Ghana; a logistic regression analysis using evidence from the demographic and health survey (1988-2014). Am J Public Heal Res.

[60] Zhu B-P, Rolfs RT, Nangle BE, Horan JM (1999). Effect of the interval between pregnancies on perinatal outcomes. N Engl J Med.

[61] Conde-Agudelo A, Belizán JM (2000). Maternal morbidity and mortality associated with interpregnancy interval: cross sectional study. BMJ..

[62] Davanzo J, Hale L, Razzaque A, Rahman M (2008). The effects of pregnancy spacing on infant and child mortality in Matlab, Bangladesh: how they vary by the type of pregnancy outcome that began the interval. Popul Stud (NY).

[63] Hehir MP, McTiernan A, Martin A, Carroll S, Gleeson R, Malone FD (2015). Improved perinatal mortality in twins-changing practice and technologies. Am J Perinatol.

[64] Townsend R, Khalil A (2018). Fetal growth restriction in twins. Best Pract Res Clin Obstet Gynaecol.

[65] Mutisya M, Kandala NB, Ngware MW, Kabiru CW (2015). Household food (in)security and nutritional status of urban poor children aged 6 to 23 months in Kenya global health. BMC Public Health.

