# Supplementary material for: Inequities in child survival in Nigerian communities during the Sustainable Development Goal era: insights from analysis of 2016/2017 Multiple Indicator Cluster Survey
Source: BMC Public Health. 2020 Oct 27;20:1613. doi: 10.1186/s12889-020-09672-8 (PMC7590598; doi:10.1186/s12889-020-09672-8)
Supplement: Supplementary file 1 — Additional file 1: Table S1. Differentials of age-specific childhood mortality rates, 2016/2017 MICS, Nigeria. [file 12889_2020_9672_MOESM1_ESM.docx]

**ADDITIONAL FILES**

**Inequities in child survival in Nigerian communities during the Sustainable Development Goal era: Insights from analysis of 2016/2017 Multiple Indicator Cluster Survey**

*Running title: Determinants of under-five mortality in Nigeria*

Daniel Adedayo Adeyinka (ORCID: 0000-0003-1855-6878),^1,2^^*^ Nazeem Muhajarine (ORCID: 0000-0001-6781-5421),^1,3^ Pammla Petruka (ORCID: 0000-0003-3174-3328)^4^ and Isaac Elon (ORCID:  0000-0002-9754-4615)^5^

^1^Department of Community Health and Epidemiology, College of Medicine, University of Saskatchewan, Saskatoon, Canada

^2^Department of Public Health, Federal Ministry of Health, Abuja, Nigeria

^3^Saskatchewan Population Health and Evaluation Research Unit, Saskatchewan, Canada

^4^College of Nursing, University of Saskatchewan, Canada

^5^Department of Paediatrics, College of Medical Sciences, Gombe State University, Nigeria

*Corresponding author: Daniel A. Adeyinka, Department of Community Health and Epidemiology, College of Medicine, University of Saskatchewan, Saskatoon, SK, S7N 5E5, Canada, Tel: +13068500086. E-mail: daa929@usask.ca

Additional Table 1: Differentials of age-specific childhood mortality rates, 2016/2017 MICS, Nigeria

| variable | Neonatal mortality rate  (per 1000 live births) | Post-neonatal mortality rate  (per 1000 live births) | Child mortality rate  (per 1000 children surviving to age 1) |
| --- | --- | --- | --- |
| **Child-level** | | | |
| Child’s sex  Male  Female | 43.2  32.4 | 30.7  26.6 | 49.7  59.1 |
| Gestation type  Singleton  Multiple | 33.7  139.0 | 27.6  56.0 | 52.9  93.1 |
| Maternal age at birth  <20 years  20-34 years  ≥35 years | 55.8  33.2  42.2 | 35.5  27.1  29.6 | 74.9  49.9  57.1 |
| Birth order  First born  2-3  4-6  ≥7 | 47.2  27.7  31.0  61.7 | 30.2  23.2  27.9  39.8 | 45.9  43.3  58.7  80.8 |
| Previous birth interval  <2 years  First birth  ≥2 years | 62.5  49.0  26.0 | 44.2  30.2  22.9 | 83.2  45.9  46.4 |
| **Maternal level** | | | |
| Maternal education  None/primary  Secondary  Post-secondary | 40.8  31.7  33.4 | 34.9  17.5  13.0 | 73.5  21.4  10.2 |
| Maternal wealth index  Poor  Middle  Rich | 39.7  42.8  32.9 | 37.5  29.0  17.4 | 81.2  54.0  19.4 |
| Mat. Media exposure  High  Medium  Low  None | 36.5  31.8  37.1  41.7 | 22.6  23.7  28.1  35.8 | 33.6  33.9  43.6  78.7 |
| Death of previous children  <3  3-4  ≥5 | 29.2  135.0  172.0 | 22.4  92.6  151 | 40.0  233.4  344.0 |
| Parity  <3  3-4  ≥5 | 33.4  31.3  45.8 | 21.4  25.8  35.4 | 40.6  43.6  69.8 |
| Access to ANC  None  Skilled  Unskilled | 39.7  36.3  55.0 | 39.5  20.4  46.9 | 81.2  47.6  86.4 |
| Freq. of ANC visits  None  1-7  ≥8 | 39.7  36.6  42.8 | 39.5  25.4  16.4 | 81.2  58.5  34.0 |
| Skilled birth attendants during delivery  None  Skilled  Unskilled | 42.6  38.4  38.2 | 36.5  16.7  35.9 | 94.5  76.9  31.4 |
| Institutional delivery  Home  Health Facilities | 38.6  38.8 | 34.2  18.0 | 80.0  29.7 |
| Contraceptive use  Yes  No | 28.2  39.1 | 23.5  31.2 | 18.8  63.1 |
| Marital status  Currently married  Formerly married  Never married | 37.6  40.3  47.4 | 28.6  31.5  20.3 | 54.1  65.1  46.2 |
| Alcohol intake  Yes  No | 34.1  38.5 | 22.2  29.5 | 26.2  59.2 |
| Smoking experience  Yes  No | 63.0  37.6 | 67.8  28.3 | 53.4  54.4 |
| **Household-level** | | | |
| Sex of household head  Male  female | 38.2  31.3 | 28.7  28.2 | 55.5  34.7 |
| Housing material index  Inadequate  Adequate | 38.8  37.3 | 36.5  23.0 | 79.8  34.7 |
| Polygamy  Yes  No | 39.8  36.9 | 34.5  25.6 | 72.4  45.0 |
| Educational status of household head  None/primary  Secondary  Post-secondary | 42.0  32.1  31.6 | 33.9  24.9  14.9 | 72.0  34.6  20.6 |
| Ethnic group of household head  Hausa  Igbo  Yoruba  Others | 40.8  28.8  35.5  35.3 | 34.9  16.9  15.8  23.5 | 82.6  21.3  16.0  32.1 |
| Household access to drinking water  Unimproved  Improved | 44.3  34.5 | 37.5  24.0 | 66.4  47.6 |
| Household sanitation  Unimproved  Improved | 38.9  36.9 | 34.4  22.7 | 62.0  45.1 |
| Indoor pollution  Polluting fuel  Clean | 38.6  27.4 | 29.5  16.0 | 57.2  8.1 |
| **Community-level** | | | |
| Place of residence  Urban  rural | 34.7  39.3 | 17.4  33.6 | 22.3  65.6 |
| Region  NC  NE  NW  SE  SS  SW | 42.5  32.7  44.1  26.2  22.0  35.6 | 26.8  26.5  37.4  20.7  18.9  15.1 | 33.1  69.9  86.2  23.5  23.7  16.3 |
| Infrastructural development  Low  High | 40.5  35.1 | 34.2  22.7 | 70.4  34.8 |
| Comm. Maternal education  Low  Medium  High | 44.2  31.4  30.3 | 38.1  19.9  16.8 | 84.7  33.5  19.8 |
